# Sex differences in left ventricular afterload and diastolic function are independent from the aortic size

**DOI:** 10.1371/journal.pone.0214907

**Published:** 2019-04-04

**Authors:** Hidemi Sorimachi, Koji Kurosawa, Kuniko Yoshida, Masaru Obokata, Takashi Noguchi, Minoru Naka, Shoichi Tange, Masahiko Kurabayashi, Kazuaki Negishi

**Affiliations:** 1 Department of Medicine and Biological Science, Gunma University Graduate School of Medicine, Maebashi, Gunma, Japan; 2 Department of Radiology, Maebashi Red Cross Hospital, Maebashi, Gunma, Japan; 3 Department of Cardiovascular Medicine, Maebashi Red Cross Hospital, Maebashi, Gunma, Japan; 4 Menzies Institute for Medical Research, University of Tasmania, Hobart, Australia; 5 Sydney Medical School Nepean, Charles Perkins Centre Nepean, University of Sydney, New South Wales, Australia; International University of Health and Welfare, School of Medicine, JAPAN

## Abstract

**Background:**

Women have a greater risk of heart failure with preserved ejection fraction (HFPEF) than men do, yet the basis for this disparity remains unclear. Greater arterial stiffness and afterload causes left ventricular (LV) diastolic dysfunction, a central mechanism of HFPEF. Because of smaller body habitus, previous reports have used body surface area as a surrogate of the size of the aorta. We performed a comprehensive hemodynamic evaluation of elderly patients with preserved EF and evaluated sex differences in the associations between LV function and afterload, before and after adjusting for the aortic sizes.

**Methods and results:**

Four hundred and forty-three patients (mean age: 73 years, 169 women) who underwent clinically indicated echocardiography and computed tomography (CT) were identified. Linear regression analyses were performed to assess the independent contributions of sex to and its interaction with LV function before and after adjusting for CT-derived aortic length and volume. Although blood pressures were similar between the sexes, women had greater arterial elastance, lower arterial compliance, and greater LV ejection fraction (all p<0.001). Sex differences were detected in the associations between LV afterload and relaxation (mitral e′) as well as in the left atrial (LA) emptying fraction, but not in LA size. These differences remained significant after adjusting for the aortic length and volume. Sensitivity analyses in an age-matched subgroup (n = 324; 162 of each sex) confirmed the robustness of these sex disparities in LV diastolic function and afterload.

**Conclusion:**

Women had worse LV relaxation than men did against the same degree of afterload, before and even after adjusting for the aortic sizes.

## Introduction

Heart failure (HF) is a major clinical and public health problem owing to its high prevalence, mortality, hospitalization, and healthcare expenditures.[1] Relative prevalence of HF with preserved ejection fraction (HFPEF) to HF with reduced EF (HFREF) is rising over time; yet the survival in HFPEF has remained dismal due to the lack of proven therapies. Several large clinical trials for HFPEF have demonstrated neutral results.[2–4] Further elucidation of the mechanisms underlying HFPEF may aid in identifying a novel therapeutic target.

Women have an approximately two-fold increased risk of developing HFPEF compared to men[5] but little is known about the precise mechanisms for this increase. Although the prevalence of HFPEF is higher in women than in men at any given age, the prevalence of HFPEF increases more rapidly with age than did the prevalence of HFREF[6]. Therefore, elderly women have a greater risk of HFPEF. Arterial stiffness augments central aortic pressure wave and increases left ventricular (LV) afterload, and thus may promote impaired diastolic function. Previous studies have shown that independent interaction of sex on the associations between afterload and diastolic functional responses after adjusting for body surface area (BSA), based on the assumption that BSA is a reasonable surrogate for the aorta size.[7, 8] Since the aorta serves both as a conduit for delivering blood to peripheral organs and as a cushion for buffering the pulsatile pressure and flow from the heart, directly measured aortic length and volume would serve as more accurate physiological indices, but there are few studies that have used such a detailed examination. We hypothesized that the sex difference in the relationships between left ventricular afterload and diastolic function would be independent from the differences in aorta size. Accordingly, the aims of this study were to confirm the sex difference in the associations between LV (systolic and diastolic) functions and afterload in our population, and then to elucidate whether these differences remain significant after adjusting for the aortic length and volume.

## Materials and methods

### Subjects

In this retrospective, cross-sectional study, all patients who had undergone clinically indicated echocardiography and computed tomography (CT) within a 12-month period were screened for eligibility. Of 848 patients who had echocardiography and CT from October 2012 to July 2014 at Maebashi Red Cross Hospital, Gunma, Japan, 405 were excluded for the following reasons: reduced LVEF (<45%) (n = 79), atrial fibrillation (n = 122), greater than mild mitral valvular heart disease (n = 28), greater than mild aortic valvular heart diseases (n = 34), LV asynergy (n = 41), heart failure (n = 47), chronic renal failure on hemodialysis (n = 53) and poor echocardiographic image quality (n = 1), leaving 443 participants for the final analyses. Their clinical indications for echocardiography were: to assess cardiac function (n = 372), to rule out infective endocarditis (n = 27) or intracardiac thrombosis (n = 29), and to assess valvular disease (= 15). The clinical indications for CT were to assess thoracic disease (n = 114), abdominal disease (n = 156), vessel disease(n = 34), hematologic disease(n = 10), fever of unknown origin(n = 36), pulmonary embolism or deep venous thrombosis(n = 16) and others(n = 77).

We also performed sensitivity analyses using 324 age-matched subjects (162 men and 162 women), in which each consecutive female subject was matched to a male participant of the same age (1:1). If an exact age match could not be found, the male subject closest in age to the female subject’s (within a 5-year difference) was selected.

The institutional medical ethics committee of the Maebashi Red Cross Hospital approved the study protocol with a waiver of the requirement to obtain informed consent (IRB number: 26–25).

### Two-dimensional and Doppler echocardiography

Two-dimensional echocardiography was performed using a commercially available ultrasound system (Vivid E9, GE healthcare, Milwaukee, Wisconsin). Standard two-dimensional measurements, including of systolic and diastolic functions, were obtained as recommended by the American Society of Echocardiography.[9, 10] Mitral inflow velocities were recorded, and the following variables were obtained: peak velocity of early diastolic mitral inflow (E), late diastolic mitral inflow (A), and the deceleration time of the E velocity. Mitral annular velocities were measured with spectral Doppler. Early diastolic mitral annular (e′), late diastolic (a′) and systolic velocities (s′) of the mitral annulus were measured from the apical 4-chamber view with a 2- to 5-mm sample volume placed at the septal and lateral corners of the mitral annulus, then averaged. The E/e′ ratio was used to estimate the LV filling pressure. Left atrial volumes (LAVs) were measured using the area-length method from the 4- and 2-chamber views and subsequently indexed (LAVI) by BSA.[11] LA emptying fraction was defined as (end-systolic LAV–end-diastolic LAV) / end-systolic LAV × 100.[12]

### Measurement of hemodynamics

The following hemodynamic parameters were estimated non-invasively. Mean arterial pressure was calculated using systolic (SBP) and diastolic blood pressures (DBP), as [(2 × DBP) + SBP] / 3. Brachial pulse pressure (PP) was calculated as brachial SBP–DBP. Total afterload was defined by the arterial elastance (Ea) (0.9 × systolic BP / SV).[13–15] The systemic vascular resistance index (SVRI), the non-pulsatile component of afterload,[16] was determined by dividing the mean BP / cardiac index × 79.9.[17] Total arterial compliance (TAC) was estimated by the ratio of SV to PP,[18] which is the change in arterial blood volume due to a given change in pulsatile arterial blood pressure.[19] Brachial-ankle pulse wave velocity (baPWV) data, using commercially available brachial artery tonometry (Omron Colin Co., Tokyo, Japan), was available in a subgroup of 100 patients and used as a parameter of arterial stiffness. The measurements were obtained in the supine position after a minimum of 5 minutes of rest.

### Measurement of aortic length and volume

Patients underwent imaging using standard commercially available CT scanners (Aquilion 64-slice; TOSHIBA or SIEMENS SOMATOM Definition Flash 128-slice; Siemens Medical Solution or SIEMENSE SOMATOM Emotion 16-slice; Siemens Medical Solution). Scanning from the neck to the pelvis was initiated in the cranio-caudal direction during a single inspiratory breath hold. Prospectively triggered axial or retrospectively gated spiral data were acquired. The tube voltage ranged from 120 to 130 kVp; tube current was adjusted by patient weight; and a beam pitch of 0.8 was used.[20–22]

**Fig 1:**
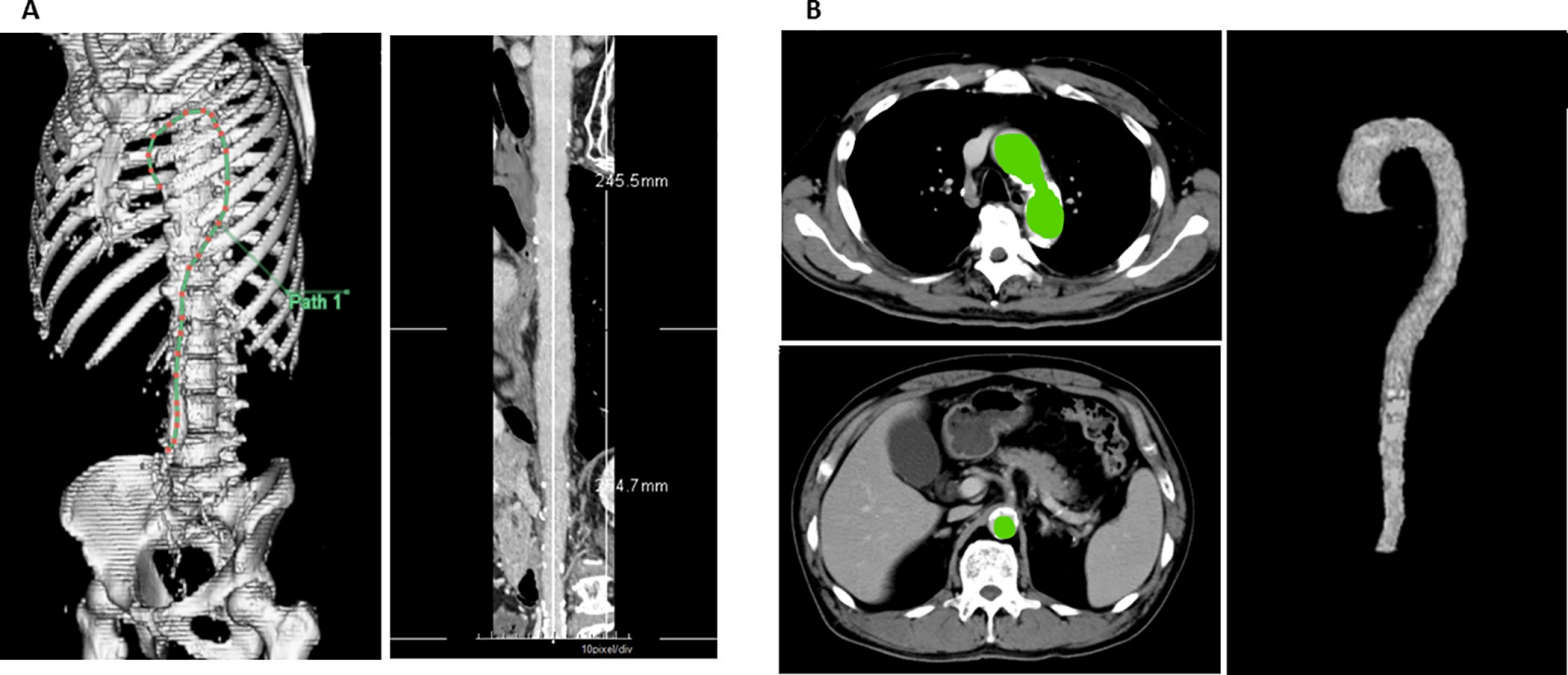
Measurement of the aortic length and volume with computed tomography. (A)The aortic length is measured by plotting the center of the vessel lumen from the ST junction to the aortic bifurcation. (B) The aortic volume is measured by tracing the arterial intima (in green) on about 20 slices from the ST junction to the aortic bifurcation on an axial-plane image, and then the volume is calculated using reconstructed data.

Subsequently, with commercially available software (Zaiostation2, Ziosoft, Tokyo, Japan), advanced offline 3-dimensional post-processing of CT images was performed using multi-planar and maximum intensity projections. Aortic length was measured by manually plotting the center of the vessel lumen from the ST junction to the aortic bifurcation (Fig 1A). Then, aortic volume was assessed by tracing the aortic intramembrane border on about 20 slices from the ST junction to the aortic bifurcation on axial-plane images and calculating the volume using reconstructed three-dimensional data (Fig 1B).

### Reproducibility of aortic length and volume

The reproducibility of the aortic length and volume measurement was assessed in 20 randomly selected patients. Intra-observer agreement was evaluated after the same observer (H.S.) repeated the measurements 4 weeks later, and inter-observer agreement was tested by comparing the measurement made by another experienced reader (M.N.). The intraclass correlation coefficients for intra-observer and inter-observer agreement for aortic length were 0.94 and 0.89. The intraclass correlation coefficients for intra-observer and inter-observer agreement for aortic volume were 0.99 and 0.98. The coefficient of variations for aortic length were 1.4% (for intra-observer) and 2.3% (for inter-observer), while those for aortic volume were 2.4% and 3.2%, respectively.

### Statistical analysis

All continuous variables are presented as mean ± standard deviation (SD) or median (25^th^ and 75^th^ percentile), as appropriate. Categorical variables are presented as absolute and relative frequencies (%). Normality was evaluated using the Shapiro-Wilk W test. e′, E/e′, and LAVI were log-transformed when put into the models based on their distributions. Differences between sexes were compared by the Student *t* test, the χ^2^ test, or the Mann-Whitney U-test, as appropriate.

To determine the associations of the indices of afterload/arteriosclerosis (SBP, mean BP, PP, Ea, SVRI, arterial compliance, aortic calcification, LV mass index, and baPWV) with LV function (log-transformed e′ velocity, E/e′, LAEF, LAVI, LVEF, and s’) for each sex, sex-specific single linear regression analyses were performed. Multivariable linear regression analyses were performed to assess the independent contributions of sex to LV functions. Then, linear regression models with an interaction term were performed to determine the effect of sex differences on the associations between afterload and LV functions or anatomy. Homoscedasticity of the errors and normality of the error distributions were checked in residual plots. We assessed whether there was a sex interaction in the relationship between afterload and LV function before and after adjusting for aortic size (aortic length and volume). A two-sided p value of <0.05 was considered statistically significant. All data were analyzed using SPSS version 21.0 (SPSS Inc., Chicago, IL, USA).

## Results

### Patient characteristics

Baseline characteristics by sex for the whole population and the age-matched population are summarized in Table 1. In the whole population, women tended to be older, but not significantly (74 vs 72, p = 0.062). All physical parameters were significantly smaller in women than in men except for body mass index. Diabetes mellitus and ischemic heart disease were more common in men. Although the serum creatinine level was higher in men, both sexes shared a similar estimated glomerular filtration rate. Approximately a half of them (n = 227) had BNP levels, where women had higher BNP than men. No differences were found in their medication use. Among the hemodynamic parameters, heart rate was higher in women than in men, although the systolic, diastolic, and mean BPs, PP, and SVRI were similar. Women had higher Ea and lower TAC than men did. These differences were also observed in the age-matched population, except for BNP level, which was similar between the sexes.

**Table 1:** Clinical characteristics of WHOLE sample and AGE-MATCED sample.

	WHOLE sample(n = 443)				AGE-MARCHED sample(n = 324)		
	Overall (n = 443)	Male (n = 274)	Female (n = 169)	p value	Male (n = 162)	Female (n = 162)	p value
Age, years	73(65, 81)	72(65, 80)	74(66, 83)	0.062	74(67, 82)	74(66, 82)	0.58
Height, cm	159±10	164±7	151±7	<0.001	163±7	151±7	<0.001
Weight, kg	57±13	61±12	50±10	<0.001	59(52, 67)	49(44, 56)	<0.001
Body surface area, m^2^	1.6±02	1.7±0.2	1.4±0.2	<0.001	1.64(1.54, 1.74)	1.43(1.34, 1.55)	<0.001
Body mass index, kg/m^2^	22.4±3.8	22.7±3.9	22.0±3.7	0.081	22.4(19.8,24.7)	21.9(1.94, 24.8)	0.48
**Comorbidities**							
Hypertension	239(54)	150(54)	89(53)	0.67	95(58)	86(53)	0.29
Diabetes mellitus	109(25)	80(29)	29(17)	0.004	49(30)	29(17)	0.009
Dyslipidemia	112(25)	78(29)	34(20)	0.050	48(29)	34(21)	0.074
Ischemic heart disease	71(17)	55(20)	16(10)	0.003	31(19)	16(10)	0.020
Smoking	207(47)	177(65)	30(18)	<0.001	105(65)	30(18)	<0.001
Hb, mg/dl	12.3±2.3	12.7±2.4	11.8(10.4,13.0)	<0.001	12.3±2.4	12.0±1.9	0.004
Creatinine, mg/dl	0.78(0.64,1.01)	0.89(0.73,1.13)	0.68(0.55,0.96)	<0.001	0.91(0.74, 1.18)	0.68(0.55, 0.86)	<0.001
eGFR, mL/min/1.73 m^2^	68.0±25.5	67.3±24.7	69.3±26.9	0.43	65.0±24.6	68.9±25.3	0.19
BNP, pg/ml†	49.2(21.7, 136.7)	39.6(18.8, 116.8)	72.5(31.2, 196.5)	0.008	48.4(22.4,136.0)	70.0(31.2, 200.2)	0.14
**Medications**							
Aspirin	67(15)	47(17)	20(12)	0.13	23(15)	18(11)	0.41
Clopidegrel	56(13)	39(14)	17(10)	0.20	29(18)	17(11)	0.062
Ca-blocker	144(32)	89(33)	55(32)	0.89	40(25)	51(32)	0.58
Beta-blockers	64(15)	43(16)	21(13)	0.36	23(14)	22(14)	0.82
ACEIs/ARBs	104(24)	63(23)	41(24)	0.78	39(24)	38(23)	0.86
Diuretics	46(10)	30(11)	16(10)	0.61	21(13)	12(8)	0.13
Statins	84(19)	59(22)	25(15)	0.073	34(21)	25(15)	0.20
**Hemodynamic Parameters**							
Heart rate, beats/min.	71(61, 81)	70(60, 79)	73(64, 82)	0.039	70(60, 79)	73(64, 83)	0.044
Systolic BP, mmHg	128(114, 141)	128(113, 140)	129(114, 144)	0.28	128(113, 141)	130(115,146)	0.20
Diastolic BP, mmHg	72(63, 83)	72(63, 83)	72(63, 83)	0.93	71(61, 82)	73(63, 84)	0.14
mean BP, mmHg	90(81, 103)	90(81, 103)	91(81, 102)	0.78	89(79, 102)	92(81, 103)	0.20
Pulse pressure, mmHg	54(45, 64)	53(44, 63)	55(46, 66)	0.18	55(45, 64)	55(46, 66)	0.79
Ea, mmHg/ml	2.6(2.1, 3.1)	2.3(1.9, 2.9)	3.0(2.5, 3.6)	<0.001	2.4(1.9, 2.9)	3.0(2.5, 3.7)	<0.001
SVRI, dyne・m^2^/s・cm^⁻5^	3580(2924, 4611)	3562(2867, 4509)	3597(3020, 4706)	0.50	3497(2819, 4452)	3642(3037, 4841)	0.17
Arterial compliance, ml/ mmHg	0.83(0.65, 1.08)	0.93(0.74, 1.17)	0.70(0.55, 0.87)	<0.001	0.91(0.73, 1.15)	0.70(0.56, 0.89)	<0.001
baPWV, cm/sec⁑	1796(1589, 2097)	1777(1536, 2037)	1872(1648, 2142)	0.26	1791(1537, 2001)	1880(1669, 2192)	0.18

Values are mean ± SD, median (interquartile range), or n (%). Hb, hemoglobin; eGFR, estimated glomerular filtration rate. BNP, brain natriuretic peptide; ACEIs/ARBs, angiotensin-converting enzyme inhibitors/angiotensin-receptor blockers; BP, blood pressure; Ea, aortic elastance; SVR, systemic vascular resistance; SVRI, systemic vascular resistance index, baPWV; brachial ankle pulse wave velocity.

†In whole population, BNP was measured in 227 cases (139 in men,88 in women).In age matched population, 165 cases(85 in men,80 in women)

⁑In whole population, baPWV was measured in 69 cases (44 in men, in 25 women). In age matched population, 49 cases(25 in men,24 in women)

### Findings from cardiovascular imaging modalities

**Fig 2:**
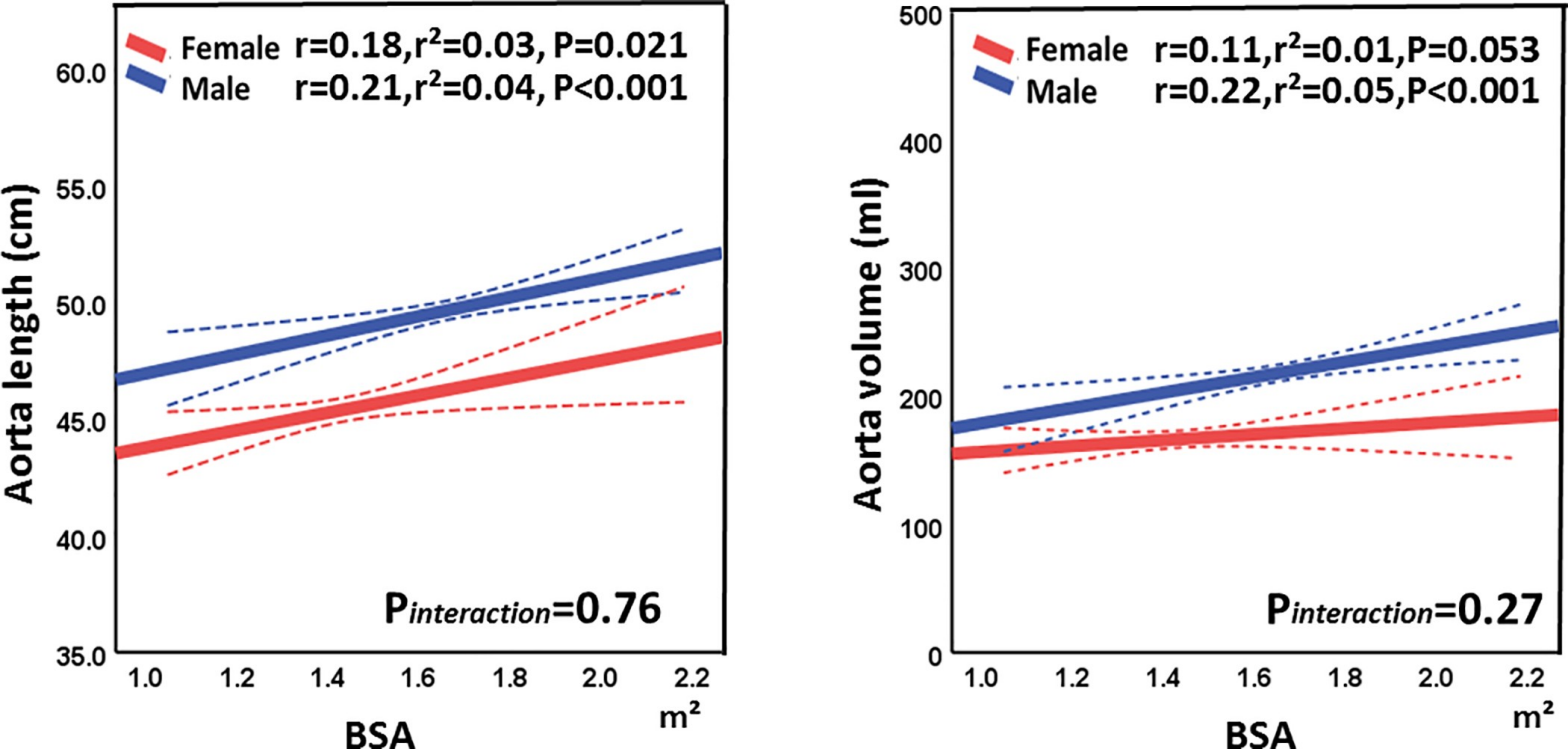
Association of aorta size and body surface area (BSA). The association with aorta length and BSA is illustrated in panel A, that with aorta volume and BSA in panel B. There were significant but weak associations between BSA and aortic sizes.

Table 2 summarizes findings from echocardiography and CT. Even after indexing to BSA, female patients had smaller LV sizes and stroke volume indices than male patients did. LVEFs were within the normal ranges but higher in women than in men. With respect to diastolic function, women had higher E/e′ ratios, slower e′s, and smaller LAEFs. The aortic length was shorter in women than in men (45.4 ± 3.4 cm vs. 49.7 ± 3.5, p<0.001). Aortic volume was smaller in women than in men (167 ± 43 ml vs. 219 ± 56 ml, p<0.001). Findings from the age-matched population were consistent with these findings. Aorta length and BSA had weak associations in both sex (women: r = 0.18, p = 0.021, men: r = 0.21, p<0.001), as well as aorta volume did (women: r = 0.11, p = 0.053, men: r = 0.22, p<0.001) (Fig 2).

**Table 2:** Findings from cardiovascular imaging modalities (echocardiography and CT).

	WHOLE sample(n = 443)				AGE-MATCHED sample(n = 324)		
	Overall (n = 443)	Men (n = 274)	Women (n = 169)	p value	Men (n = 180)	Women (n = 180)	p value
**Echocardiographic Findings**							
Septal wall thickness, mm	9.5±2.1	9.7±2.1	9.0(7.9, 10.6)	0.022	10.0±2.4	9.3±2.0	0.009
Posterior wall thickness, mm	9.4±1.5	9.6±1.4	9.1(8.1, 10.2)	<0.001	9.8±2.3	9.1±1.5	0.002
LV mass index, g/m^2^	86.2(71.3, 102.5)	89.3	84.9	0.22	86.8(74.5, 102.0)	83.8(68.9, 100.3)	0.20
LV end-diastolic volume index, ml/ m^2^	44.8(38.2, 54.8)	47.8(39.7, 56.7)	41.6(36.5, 49.6)	<0.001	48.4(39.8, 57.2)	41.5(36.3, 49.2)	<0.001
LV end-systolic volume index, ml/ m^2^	16.6(12.9, 21.5)	17.8(14.1, 22.3)	14.3(11.3, 18.7)	<0.001	17.4(14.1, 22.4)	14.3(11.3, 18.6)	<0.001
LVEF, %	63.4±6.4	62.5±6.6	64.8±5.8	<0.001	62.5±6.6	64.6±5.7	0.002
Stroke volume index, ml/ m^2^	28.5(24.2, 34.0)	29.3(24.9, 34.6)	27.2(23.3, 31.8)	0.008	29.6(24.9, 35.4)	27.0(23.0, 31.5)	0.007
E velocity, cm/sec	60.2(51.2, 71.0)	58.6(50.7, 69.6)	62.3(52.8, 73.8)	0.049	60.2(50.3, 71.1)	62.3(52.2, 72.2)	0.33
A velocity, cm/sec	81.9±22.1	78.6±21.1	87.4±22.6	<0.001	79.6±20.4	87.2±21.5	0.001
E/A ratio	0.75(0.62, 0.90)	0.76(0.64, 0.90)	0.72(0.60, 0.91)	0.12	0.72(0.62, 0.92)	0.72(0.60, 0.90)	0.19
Deceleration time, msec	246±68	246±67	246±70	0.95	247±74	246±69	0.87
e’(average), cm/sec	6.4(5.3, 7.9)	6.6(5.5, 8.2)	6.1(4.9, 7.7)	0.004	6.5(5.2, 7.9)	6.1(4.9, 7.7)	0.10
a’(average), cm/sec	9.6±2.1	9.7±2.0	9.3±2.1	0.045	9.5±2.1	9.4±2.1	0.75
s’(average), cm/sec	7.6±2.0	7.8±2.1	7.2±1.8	0.001	7.7±2.0	7.3±1.7	0.034
E/e’(average)	9.1(7.3, 11.8)	8.8(7.0, 11.2)	9.8(7.8, 12.8)	<0.001	9.3(7.1, 11.5)	9.7(7.8, 12.6)	0.033
LAVI max, mL/m^2^	28.0(22.1, 36.9)	27.2(21.5, 35.4)	28.6(22.5, 38.6)	0.082	28.8(21.9, 36.5)	28.4(22.5, 37.5)	0.63
LAVI min, mL/ m^2^	12.0(8.1, 17.1)	11.7(7.5, 16.3)	13.1(9.5, 19.5)	0.002	11.9(7.5, 16.5)	12.8(9.6, 19.4)	0.020
LAEF, %	57.2(48.1, 65.9)	58.8(49.4, 68.3)	55.5(46.4, 61.5)	<0.001	60.6(48.8, 68.9)	55.6(46.5, 61.7)	<0.001
**CT parameters**							
Aorta length, cm	48.1±4.1	49.7±3.5	45.4±3.4	<0.001	49.7±3.5	45.5±3.4	<0.001
Volume of aorta, ml	199±58	219±56	167±43	<0.001	222±59	167±43	<0.001

Values are mean ± SD or median (interquartile range). E, early mitral diastolic inflow velocity; e’, early diastolic mitral annular velocity; LAEF, left atrial emptying fraction; LAVI max, maximum left atrial volume index; LAVI min, minimum left atrial volume index; LV, left ventricular; LVEF, left ventricular ejection fraction; a’, end diastolic velocity of the mitral annulus; s’, systolic mitral annular velocity.

### Sex differences in the associations between afterload parameters and cardiac functions

**Fig 3:**
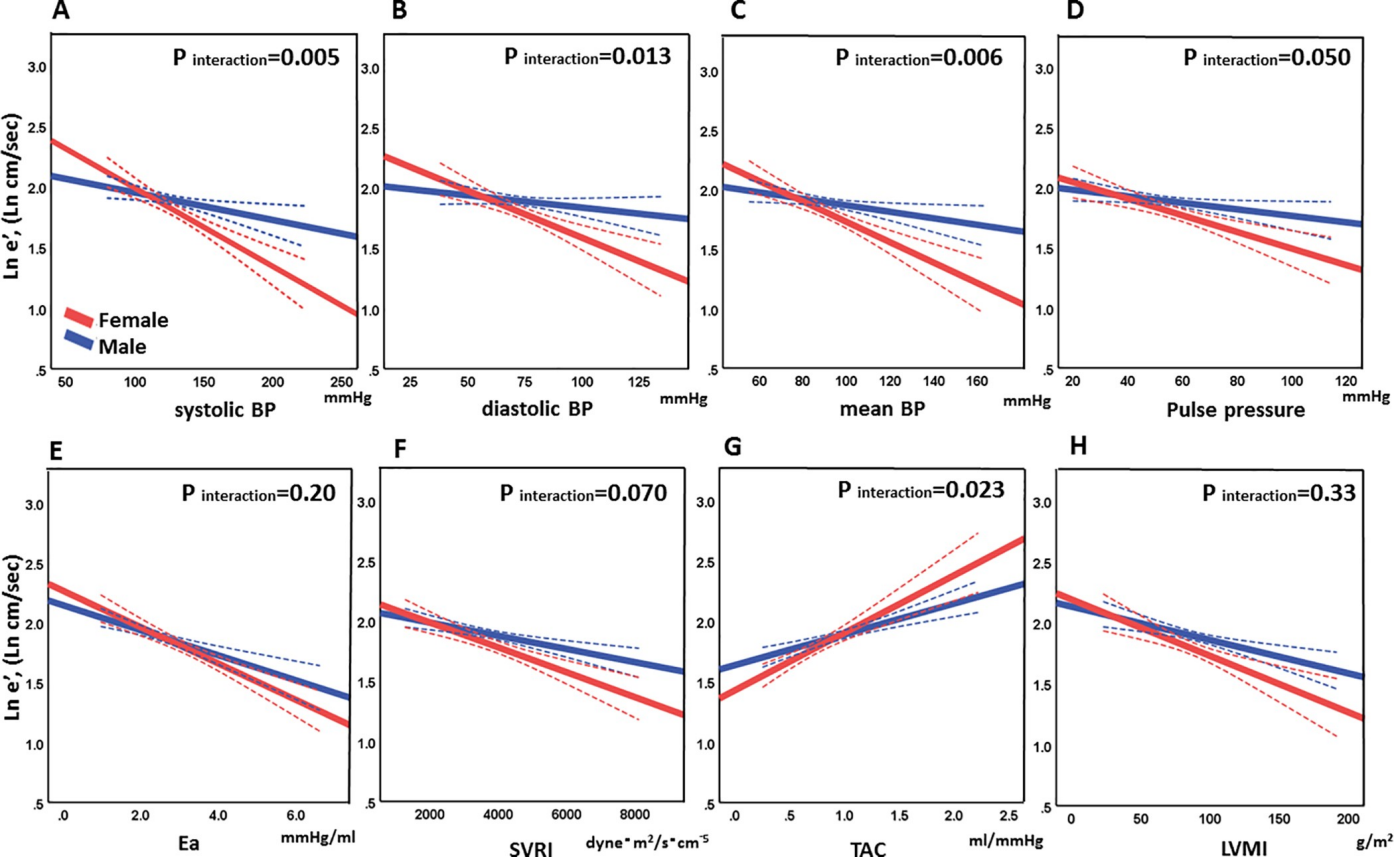
Interactions by sex for the associations between ln e′ and afterload in the whole population. The association of the log-transformed e′ against systolic blood pressure (BP) is illustrated in A, against diastolic BP in B, against mean BP in C, against pulse pressure in D, against Ea in E, against SVRI in F, against TAC in G, and against LVMI in H. Ea, arterial elastance; LVMI, left ventricular mass index; SVRI, systemic vascular resistance index; TAC, total arterial compliance.

Sex-specific associations between the afterload markers and cardiac anatomy or mechanics in the whole population are summarized in Table 3. Significant sex differences were confirmed in the associations between e′ and the indices of afterload, except for Ea (Fig 3), where women had lower e’ than men to the similar level of afterload. These differences remained significant after adjusting for aortic length and volume except for SVRI. Significant interactions were also noted between E/e’ and systolic or mean BP, even after adjusting for the aortic length or volume. Among LA parameters, interactions were observed between LA function (i.e. LAEF) and BPs but not between LA volume and afterload markers. Intriguingly, significant associations between LV systolic function (i.e. LVEF) and several afterload parameters (diastolic BP, mean BP, Ea, SVRI, and TAC) were seen only in men but none in women. Then, sex differences were seen in the relationships between LVEF and Ea or SVRI, which remained significant after adjusting for the aortic sizes. Collectively, LV diastolic function and afterload parameters had stronger association in women than men even after adjusting for the aorta sizes. Whereas men had stronger association between LV systolic function and afterload than women, regardless of adjusting for the aortic sizes or not.

**Table 3:** Sex specific linear regression analysis and sex differences in associations between afterload and cardiac function in WHOLE sample.

	Univariable linear regression models				Models with interaction for sex difference					
	Men (n = 307)		Women (n = 189)		Interaction		Interaction Adjusted for Aorta length		Interaction Adjusted for Aorta volume	
	β(95% CI)	p value	β(95% CI)	p value	β	p-value	β	p-value	β	p-value
Ln e’(average), cm/sec										
systolic BP, mmHg	-0.002(-0.004, -0.001)	0.011	-0.006(-0.009, -0.004)	<0.001	-0.004	0.003	-0.004	0.005	-0.004	0.023
diastolic BP, mmHg	-0.002(-0.005, 0.001)	0.12	-0.008(-0.011, -0.004)	<0.001	-0.006	0.007	-0.005	0.013	-0.006	0.008
mean BP, mmHg	-0.003(-0.005, 0.000)	0.021	-0.009(-0.012, -0.005)	<0.001	-0.006	0.003	-0.005	0.006	-0.006	0.004
Pulse pressure, mmHg	-0.003(-0.005, 0.000)	0.035	-0.007(-0.010, -0.004)	<0.001	-0.004	0.041	-0.004	0.050	-0.004	0.035
Ea, mmHg/ml	-0.11(-0.149, -0.060)	<0.001	-0.15(-0.199, -0.105)	<0.001	-0.047	0.15	-0.041	0.20	-0.042	0.18
SVRI, dyne・m^2^/s・cm^⁻5^	-0.006(-0.008,0.003)	<0.001	-0.010(-0.014, -0.006)	<0.001	-0.005	0.043	-0.004	0.070	-0.004	0.059
TAC, ml/mmHg	0.26(0.154, 0.357)	<0.001	0.48(0.313, 0.642)	<0.001	0.22	0.021	0.21	0.023	0.21	0.023
LVMI, g/ m^2^	-0.002(-0.004, 0.000)	0.013	-0.004(-0.006, -0.002)	<0.001	-0.002	0.15	-0.001	0.33	-0.002	0.18
Ln baPWV, cm/sec⁑	-0.44(-0.912, 0.027)	0.064	-0.74(-1.384, -0.087)	0.028	-0.29	0.44	-0.11	0.77	-0.21	0.81
Ln E/e’(average)										
systolic BP, mmHg	0.002(0.000, 0.004)	0.068	0.005(0.003, 0.008)	<0.001	0.003	0.038	0.003	0.043	0.003	0.041
diastolic BP, mmHg	-0.001(-0.004, 0.002)	0.44	0.003(-0.001, 0.007)	0.16	0.004	0.10	0.004	0.12	0.004	0.11
mean BP, mmHg	0.001(-0.002, 0.003)	0.45	0.006(0.002, 0.009)	0.004	0.005	0.029	0.005	0.034	0.005	0.032
Pulse pressure, mmHg	0.005(0.002, 0.008)	0.001	0.008(0.004, 0.012)	<0.001	0.003	0.16	0.003	0.16	0.004	0.13
Ea, mmHg/ml	0.027(-0.026, 0.079)	0.32	0.082(0.023, 0.140)	0.006	0.055	0.16	0.052	0.18	0.053	0.17
SVRI, dyne・m^2^/s・cm^⁻5^	0.001(-0.002, 0.004)	0.51	0.004(0.001, 0.009)	0.004	0.003	0.33	0.002	0.41	0.003	0.36
TAC, ml/mmHg	-0.14(-0.257, -0.019)	0.023	-0.33(-0.531, -0.130)	0.001	-0.19	0.098	0.19	0.10	-0.19	0.10
LVMI, g/ m^2^	0.003(0.001, 0.005)	0.001	0.004(0.001, 0.006)	0.002	0.00	0.91	-0.001	0.95	0.00	0.94
Ln baPWV, cm/sec⁑	0.45(0.010, 0.879)	0.045	0.85(0.346, 1.354)	0.002	0.41	0.22	0.45	0.77	0.39	0.31
Ln LAVI max, ml/ m^2^										
systolic BP, mmHg	0.002(0.000, 0.004)	0.042	0.004(0.001, 0.006)	0.002	0.002	0.26	0.002	0.27	0.002	0.49
diastolic BP, mmHg	-0.002(-0.006, 0.001)	0.13	0.000(-0.004, 0.004)	0.84	0.002	0.43	0.002	0.48	0.002	0.39
mean BP, mmHg	0.000(-0.003, 0.003)	0.99	0.002(-0.001, 0.006)	0.22	0.002	0.33	0.002	0.36	0.002	0.34
Pulse pressure, mmHg	0.007(0.004, 0.010)	<0.001	0.009(0.006, 0.012)	<0.001	0.002	0.35	0.002	0.36	0.002	0.37
Ea, mmHg/ml	-0.061(-0.116, -0.005)	0.013	-0.046(-0.103, 0.010)	0.11	0.014	0.72	0.012	0.77	0.009	0.83
SVRI, dyne・m^2^/s・cm^⁻5^	-0.002(-0.006, 0.001)	0.032	-0.004(-0.008, 0.001)	0.14	-0.001	0.67	-0.002	0.58	-0.002	0.53
TAC, ml/mmHg	0.011(-0.114, 0.136)	0.96	-0.050(-0.246, 0.146)	0.61	-0.047	0.69	-0.044	0.71	-0.034	0.78
LVMI, g/ m^2^	0.008(0.006, 0.009)	<0.001	0.007(0.005, 0.010)	<0.001	0.001	0.34	0.001	0.29	0.001	0.32
Ln baPWV, cm/sec⁑	0.088(-0.396, 0.573)	0.72	0.35(0.162, 0.856)	0.17	0.26	0.46	0.42	0.18	0.27	0.30
LAEF, %										
systolic BP, mmHg	0.034(-0.047, 0.115)	0.95	-0.16(-0.247, -0.073)	<0.001	-0.19	0.002	-0.20	0.001	-0.19	0.002
diastolic BP, mmHg	-0.010(-0.109, 0.130)	0.82	-0.13(-0.263, 0.010)	0.070	-0.14	0.15	-0.14	0.13	-0.14	0.14
mean BP, mmHg	0.033(-0.075, 0.140)	0.98	-0.16(-0.285, -0.030)	0.016	-0.19	0.028	-0.20	0.023	-0.19	0.029
Pulse pressure, mmHg	0.065(-0.052, 0.182)	0.73	-0.23(-0.357, -0.108)	<0.001	-0.30	0.001	-0.30	0.001	-0.31	0.001
Ea, mmHg/ml	-1.38(-3.50, 0.74)	0.39	-2.27(-4.210, -0.330)	0.022	-0.90	0.55	-0.98	0.51	0.88	0.56
SVRI, dyne・m^2^/s・cm^⁻5^	-0.13(-0.252, 0.002)	0.10	-0.16(-0.319, 0.007)	0.006	-0.031	0.77	-0.049	0.64	-0.031	0.77
TAC, ml/mmHg	0.59(-3.540, 6.232)	0.74	5.13(-1.655, 11.84)	0.014	3.75	0.39	3.69	0.39	3.64	0.41
LVMI, g m^2^	-0.070(-0.138, -0.002)	0.003	-0.11(-0.190, -0.017)	0.020	-0.034	0.56	-0.041	0.47	-0.037	0.52
Ln baPWV, cm/sec⁑	-3.87(-21.39, 13.66)	0.66	-11.1(-31.59, 9.448)	0.28	1.12	0.95	-6.21	0.73	-0.73	0.99
LVEF, %										
systolic BP, mmHg	-0.032(-0.069, 0.005)	0.091	0.012(-0.027, 0.051)	0.55	0.044	0.12	0.044	0.12	0.049	0.85
diastolic BP, mmHg	-0.067(-0.122,-0.012)	0.016	0.011(-0.048, 0.071)	0.71	0.078	0.063	0.078	0.066	0.077	0.065
mean BP, mmHg	-0.058(-0.108, -0.009)	0.020	0.008(-0.048, 0.064)	0.78	0.067	0.088	0.066	0.092	0.069	0.074
Pulse pressure, mmHg	0.001(-0.050, 0.053)	0.96	0.021(-0.028, 0.070)	0.41	0.022	0.59	0.021	0.60	0.036	0.38
Ea, mmHg/ml	-1.77(-2.735, -0.813)	<0.001	-0.25(-1.099, 0.604)	0.57	1.53	0.021	1.53	0.021	1.66	0.012
SVRI, dyne・m^2^/s・cm^⁻5^	-0.12(-0.173, -0.058)	<0.001	-0.021(-0.092, 0.050)	0.56	0.095	0.048	0.096	0.045	0.11	0.026
TAC, ml/mmHg	2.98(-0.747, 5.209)	0.009	1.02(-1.909, 3.950)	0.69	-1.96	0.31	-1.92	0.32	-2.33	0.23
LVMI, g/ m^2^	-0.03(-0.062, 0.001)	0.056	-0.013(-0.051, 0.025)	0.51	0.018	0.49	0.016	0.34	0.025	0.35
Ln baPWV, cm/sec⁑	-0.278(-11.95, 6.387)	0.55	1.067(-7.159, 9.293)	0.79	-1.06	0.89	-2.22	0.62	-0.41	0.92
s’,cm/sec										
systolic BP, mmHg	-0.013(-0.025, -0.001)	0.028	-0.019(-0.030, -0.007)	0.002	-0.006	0.51	-0.006	0.49	-0.006	0.61
diastolic BP, mmHg	-0.008(-0.025, 0.010)	0.39	-0.015(-0.033, 0.003)	0.099	-0.008	0.57	-0.008	0.55	-0.008	0.57
*mean BP*, *mmHg*	-0.014(-0.030, 0.001)	0.076	-0.023(-0.040, -0.006)	0.008	-0.009	0.47	-0.009	0.45	-0.009	0.48
Pulse pressure, mmHg	0.001(-0.050, 0.053)	0.96	0.021(-0.028, 0.070)	0.41	-0.006	0.64	-0.006	0.67	-0.007	0.57
Ea, mmHg/ml	-0.52(-0.826, -0.221)	0.001	-0.33(-0.588, -0.078)	0.38	0.191	0.35	0.19	0.36	0.18	0.37
SVRI, dyne・m^2^/s・cm^⁻5^	-0.045(-0.063, -0.027)	<0.001	-0.033(-0.054, -0.012)	0.011	0.012	0.42	0.011	0.11	0.010	0.50
TAC, ml/mmHg	1.24(0.546, 1.936)	0.001	1.33(0.462, 2.204)	0.003	0.092	0.88	0.098	0.87	0.11	0.85
LVMI, g/ m^2^	-0.014(-0.024, -0.004)	0.006	-0.018(-0.029, -0.007)	0.002	-0.004	0.17	-0.005	0.52	-0.004	0.59
Ln baPWV, cm/sec⁑	-0.278(-11.95, 6.387)	0.55	1.067(-7.159, 9.293)	0.79	0.021	0.99	-0.055	0.98	0.021	0.58

e’, end diastolic velocity of the mitral annulus; LAVI max, maximum left atrial volume index; LAEF, left atrial emptying fraction; LVEF, left ventricular ejection fraction; s’, systolic mitral annular velocity; BP, blood pressure; Ea, arterial elastance; SVRI, systemic vascular resistance index; TAC, total arterial compliance; LVMI, left ventricular mass index; Ln baPWV, log-transformed brachial ankle pulse wave velocity.

⁑In whole population, baPWV was measured in 69 cases (44 in men, in 25 women). In age matched population, 49 cases (25 in men, 24 in women)

**Fig 4:**
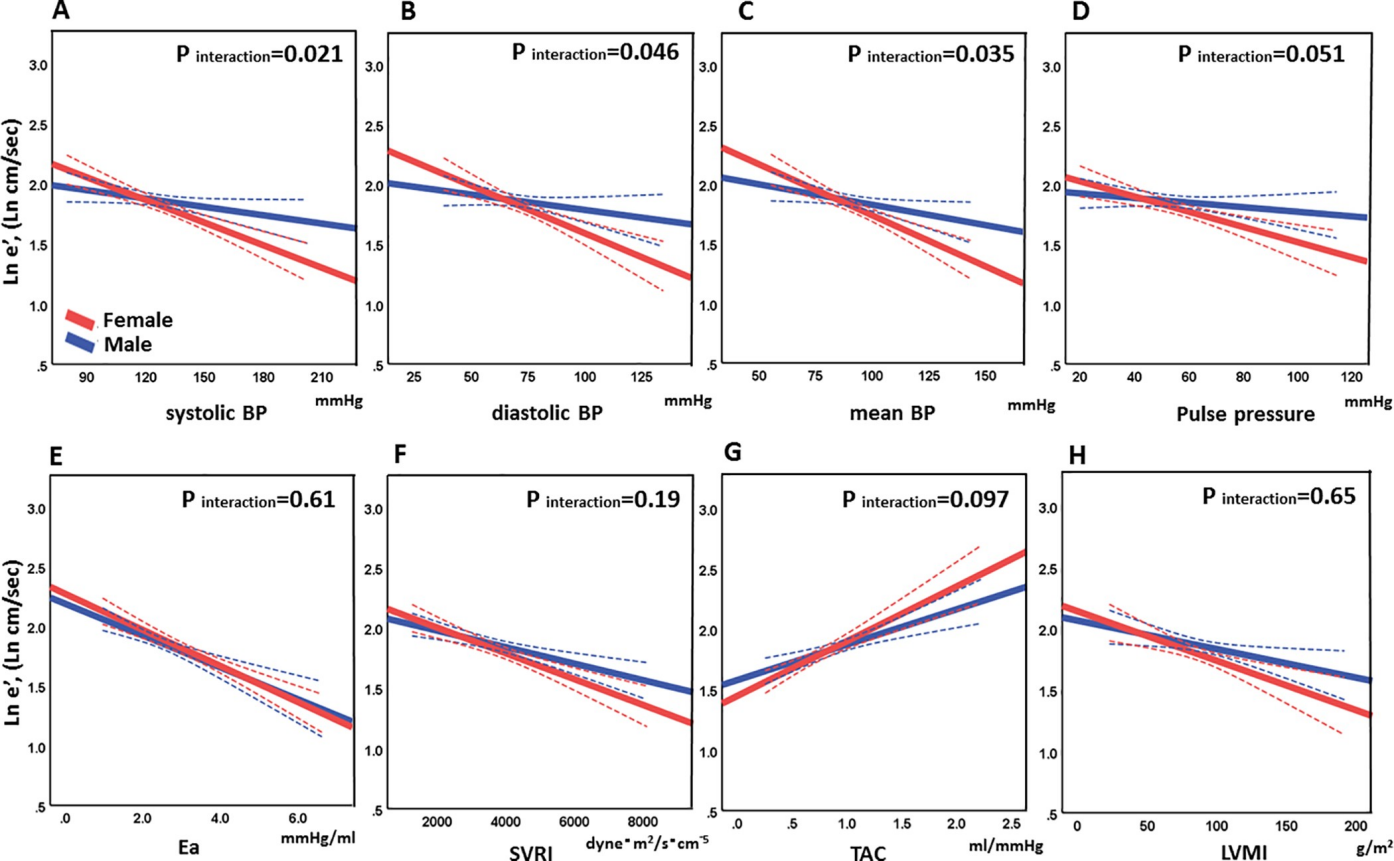
Interactions by sex for the associations between ln e′ and afterload in the age-matched population. The association of the log-transformed e′ against systolic blood pressure (BP) is illustrated in A, against diastolic BP in B, against mean BP in C, against pulse pressure in D, against Ea in E, against SVRI in F, against TAC in G, and against LVMI in H. Abbreviations are in Fig 2.

The results from the sensitivity analyses in the age-matched population are reported in Table 4 and Fig 4. In general, findings from this subgroup corroborated above findings from the whole population. Sex differences observed in the age-matched cohort were in the associations between e′ and BPs (p<0.05 for BPs with borderline significance in pulse pressure, p = 0.051 ~ 0.053), but not with SVRI and TAC, irrespective of adjustment for the aortic sizes. Of note, the independent sex difference between E/e′ and diastolic BP after adjusting for the aortic length was confirmed in this age-matched cohort (p = 0.049), but with borderline significance after adjusting for the aortic volume (p = 0.051). Sex differences between LAEF and BPs remained significant, but not those with other afterload markers. No sex differences between LA size and afterload were corroborated. Regarding LV systolic function, the sensitivity analyses supported all of the findings in the whole population. Significant sex differences between LVEF and afterload parameters were found, in which there was an inverse linear association between LVEF and Ea only in men but not in women, with a significant sex interaction before and after adjusting for the aortic length (p = 0.018 and 0.017, respectively) and for the aortic volume (p = 0.010). Similar sex differences were found in the relationship between LVEF and SVRI before (p = 0.021) and after adjusting for the aorta sizes (aorta length; p = 0.020, aorta volume; p = 0.008). These finding were also confirmed in the models adjusted for height and weight (Table 5)

**Table 4:** Sex specific univariable linear regression analysis and sex differences in associations between afterload and cardiac function in AGE-MATCHED sample.

	Univariable linear regression models				Models with interaction for sex difference					
	Men (n = 162)		Women (n = 162)		Interaction		Interaction Adjusted for Aorta length		Interaction Adjusted for Aorta volume	
	β(95% CI)	p value	β(95% CI)	p value	β	p-value	β	p-value	β	p-value
Ln e’(average), cm/sec										
systolic BP, mmHg	-0.002(-0.005, 0.000)	0.052	-0.006(-0.008, -0.004)	<0.001	-0.004	0.014	-0.004	0.021	-0.004	0.017
diastolic BP, mmHg	-0.003(-0.006, 0.001)	0.14	-0.008(-0.011, -0.005)	<0.001	-0.005	0.027	-0.005	0.046	-0.005	0.029
mean BP, mmHg	-0.003(-0.007, 0.000)	0.032	-0.009(-0.012, -0.005)	<0.001	-0.004	0.022	-0.005	0.035	-0.005	0.025
Pulse pressure, mmHg	-0.002(-0.005, 0.001)	0.24	-0.006(-0.010, -0.003)	<0.001	-0.004	0.053	-0.004	0.051	-0.004	0.051
Ea, mmHg/ml	-0.13(-0.189, -0.078)	<0.001	-0.15(-0.196, -0.107)	<0.001	-0.018	0.62	-0.018	0.61	-0.013	0.70
SVRI, dyne・m^2^/s・cm^⁻5^	-0.007(-0.010, -0.004)	<0.001	-0.010(-0.013, -0.006)	<0.001	-0.004	0.14	-0.003	0.19	-0.003	0.19
TAC, ml/mmHg	0.29(0.154, 0.433)	<0.001	0.45(0.333, 0.646)	<0.001	0.16	0.13	0.17	0.097	0.16	0.12
LVMI, g/ m^2^	-0.002(-0.004, 0.000)	0.017	-0.004(-0.006, -0.002)	<0.001	-0.002	0.25	-0.001	0.65	-0.001	0.39
Ln baPWV, cm/sec⁑	-0.92(-1.738, -0.105)	0.029	-0.35(-1.074, -0.384)	0.34	0.58	0.29	0.60	0.27	0.00	0.25
Ln E/e’(average)										
systolic BP, mmHg	0.003(0.000, 0.005)	0.031	0.005(0.003, 0.008)	<0.001	0.002	0.19	0.002	0.20	0.002	0.22
diastolic BP, mmHg	-0.001(-0.005, 0.003)	0.60	0.005(0.001, 0.008)	0.028	0.006	0.044	0.005	0.049	0.005	0.051
mean BP, mmHg	0.001(-0.002, 0.005)	0.39	0.006(0.003, 0.010)	0.001	0.002	0.061	0.005	0.065	0.005	0.063
Pulse pressure, mmHg	0.006(0.002, 0.009)	0.001	0.007(0.003, 0.010)	<0.001	0.001	0.79	0.001	0.78	0.001	0.72
Ea, mmHg/ml	0.046(-0.018, 0.109)	0.16	0.091(0.037, 0.145)	0.001	0.046	0.78	0.045	0.29	0.040	0.35
SVRI, dyne・m^2^/s・cm^⁻5^	0.002(-0.002, 0.006)	0.26	0.005(0.000, 0.010)	0.032	0.003	0.34	0.003	0.37	0.002	0.45
TAC, ml/mmHg	-0.15(-0.226, -0.015)	0.015	-0.31(-0.498, -0.127)	0.001	-0.12	0.33	-0.12	0.31	-0.11	0.38
LVMI, g/ m^2^	0.003(0.001, 0.005)	0.003	0.003(0.001, 0.006)	0.014	0.001	0.99	0.00	0.98	0.001	0.39
Ln baPWV, cm/sec⁑	0.69(0.045, 1342)	0.037	0.22(0.446, 0.891)	0.50	-0.47	0.31	-0.45	0.34	0.00	0.25
Ln LAVI max, ml/ m^2^										
systolic BP, mmHg	0.003(0.001, 0.005)	0.005	0.004(0.001, 0.006)	0.002	0.00	0.85	0.00	0.86	0.00	0.89
diastolic BP, mmHg	-0.002(-0.005, 0.001)	0.21	-0.001(-0.005, 0.003)	0.65	0.004	0.23	0.004	0.24	0.004	0.19
mean BP, mmHg	0.001(-0.002, 0.004)	0.44	0.002(-0.002, 0.005)	0.41	0.00	0.36	0.003	0.37	0.003	0.35
Pulse pressure, mmHg	0.008(0.005, 0.010)	<0.001	0.008(0.005, 0.011)	<0.001	-0.001	0.59	-0.001	0.59	-0.002	0.55
Ea, mmHg/ml	-0.068(-0.121, -0.014)	0.013	-0.055(-0.109, -0.002)	0.044	0.008	0.87	0.007	0.88	0.003	0.94
SVRI, dyne・m^2^/s・cm^⁻5^	-0.003(-0.006, 0.000)	0.092	-0.004(-0.009, 0.000)	0.037	-0.002	0.62	-0.002	0.56	-0.002	0.50
TAC, ml/mmHg	0.011(-0.114, 0.136)	0.86	-0.047(-0.243, 0.148)	0.63	0.081	0.56	0.078	0.57	0.091	0.51
LVMI, g/ m^2^	0.008(0.006, 0.009)	<0.001	0.007(0.005, 0.010)	<0.001	0.00	0.76	0.001	0.65	0.001	0.73
Ln baPWV, cm/sec⁑	0.47(-0.135, 1.080)	0.12	0.48(-0.157, 1.110)	0.13	0.30	0.56	0.34	0.49	0.00	0.56
LAEF, %										
systolic BP, mmHg	0.002(-0.072, 0.077)	0.95	-0.16(-0.240, -0.079)	<0.001	-0.22	0.002	-0.22	0.002	-0.22	0.002
diastolic BP, mmHg	-0.013(-0.125, 0.099)	0.82	-0.11(-0.231, 0.022)	0.11	-0.20	0.058	-0.21	0.048	-0.21	0.053
mean BP, mmHg	0.001(-0.098, 0.101)	0.98	-0.145(-0.266, -0.024)	0.019	-0.22	0.011	-0.26	0.009	-0.26	0.011
Pulse pressure, mmHg	0.019(-0.087, 0.125)	0.73	-0.21(-0.320, -0.104)	<0.001	-0.28	0.005	-0.28	0.005	-0.29	0.004
Ea, mmHg/ml	-0.86(-2.847, 1.123)	0.39	-0.002(-0.003, 0.000)	0.004	-1.33	0.43	-1.38	0.41	-1.33	0.44
SVRI, dyne・m^2^/s・cm^⁻5^	-0.001(-0.002, 0.000)	0.10	6.24(-0.249, 12.735)	0.018	-0.037	0.76	-0.056	0.64	-0.039	0.75
TAC, ml/mmHg	0.79(-3.781, 5.356)	0.74	-0.067(-0.202, 0.068)	0.059	2.68	0.58	2.50	0.61	2.58	0.60
LVMI, g m^2^	0.20(0.031, 0.371)	0.021	0.21(-0.042, 0.464)	0.10	0.001	0.99	-0.009	0.88	-0.003	0.96
Ln baPWV, cm/sec⁑	5.0(-23.25, 33.21)	0.72	-10.4(-35.48, 14.61)	0.40	-14.2	0.60	-16.0	0.54	-0.010	0.54
LVEF, %										
systolic BP, mmHg	-0.026(-0.077, 0.024)	0.31	0.016(-0.024, 0.055)	0.44	0.042	0.20	0.042	0.20	0.048	0.13
diastolic BP, mmHg	-0.056(-0.129, 0.016)	0.13	0.017(-0.044, 0.078)	0.58	0.074	0.13	0.073	0.13	0.075	0.12
mean BP, mmHg	-0.050(-0.118, 0.017)	0.14	0.014(-0.044, 0.071)	0.64	0.042	0.15	0.064	0.16	0.067	0.13
Pulse pressure, mmHg	-0.002(-0.071, 0.067)	0.95	0.020(-0.036, 0.076)	0.49	0.022	0.62	0.021	0.63	0.037	0.41
Ea, mmHg/ml	-1.96(-3.12, -0.739)	0.002	-0.18(-1.040, 0.675)	0.68	-1.78	0.018	1.79	0.017	1.92	0.010
SVRI, dyne・m^2^/s・cm^⁻5^	-0.13(-0.205, -0.061)	<0.001	-0.012(-0.085, 0.061)	0.75	0.12	0.021	0.12	0.020	0.14	0.008
TAC, ml/mmHg	3.92(0.851, 6.980)	0.013	0.97(-1.976, 3.912)	0.52	-2.95	0.18	-2.94	0.18	-3.21	0.14
LVMI, g/ m^2^	-0.050(-0.091, -0.009)	0.018	-0.012(-0.050, 0.027)	0.55	0.038	0.19	0.036	0.22	0.049	0.089
Ln baPWV, cm/sec⁑	4.32(-17.11, 25.74)	0.68	0.24(-10.88, 11.37)	0.96	-4.07	0.72	-4.30	0.71	-0.002	0.78
s’, cm/sec										
systolic BP, mmHg	-0.012(-0.027, 0.003)	0.11	-0.018(-0.029, -0.006)	0.002	-0.006	0.55	-0.006	0.53	-0.006	0.54
diastolic BP, mmHg	-0.007(-0.029, 0.014)	0.51	-0.017(-0.035, 0.000)	0.053	-0.010	0.075	-0.011	0.46	-0.011	0.46
mean BP, mmHg	-0.014(-0.034, 0.006)	0.17	-0.024(-0.040, -0.007)	0.005	-0.006	0.47	-0.010	0.44	-0.010	0.46
Pulse pressure, mmHg	-0.015(-0.035, 0.005)	0.15	-0.021(-0.037, -0.005)	0.010	-0.006	0.64	-0.006	0.63	-0.007	0.59
Ea, mmHg/ml	-0.61(-0.970, -0.240)	0.001	-0.33(-0.576, -0.085)	0.009	0.28	0.21	0.28	0.21	0.26	0.23
SVRI, dyne・m^2^/s・cm^⁻5^	-0.044(-0.065, -0.022)	<0.001	-0.034(-0.054, -0.013)	0.001	0.010	0.16	0.009	0.55	0.008	0.62
TAC, ml/mmHg	1.37(0.456, 2.279)	0.004	1.15(0.305, 1.991)	0.008	-0.22	0.73	-0.22	0.73	-0.19	0.77
LVMI, g/ m^2^	-0.022(-0.034, -0.010)	0.001	-0.014(-0.025, -0.003)	0.016	0.008	0.34	0.007	0.43	0.008	0.36
Ln baPWV, cm/sec⁑	-316(-8.650, 2.329)	0.25	0.39(-1.867, 2.647)	0.72	3.55	0.20	3.41	0.21	0.002	0.22

e’, end diastolic velocity of the mitral annulus; LAVI max, maximum left atrial volume index; LAEF, left atrial emptying fraction; LVEF, left ventricular ejection fraction; s’, systolic mitral annular velocity; BP, blood pressure; Ea, arterial elastance; SVRI, systemic vascular resistance index; TAC, total arterial compliance; LVMI, left ventricular mass index; Ln baPWV, log-transformed brachial ankle pulse wave velocity.

⁑In whole population, baPWV was measured in 69 cases (44 in men, in 25 women). In age matched population, 49 cases (25 in men, 24 in women)

**Table 5:** Sex specific linear regression analysis and sex differences in associations between afterload and cardiac function in WHOLE sample.

	Linear regression models				Models with interaction for sex difference					
	Men (n = 307)		Women (n = 189)		Interaction		Interaction Adjusted for Aorta length		Interaction Adjusted for Aorta volume	
	β(95% CI)	p value	β(95% CI)	p value	β	p-value	β	p-value	β	p-value
Ln e’(average), cm/sec										
systolic BP, mmHg	-0.002(-0.003, 0.000)	0.071	-0.006(-0.008, -0.004)	<0.001	-0.004	0.001	-0.004	0.002	-0.005	<0.001
diastolic BP, mmHg	-0.002(-0.005, 0.001)	0.18	-0.008(-0.011, -0.005)	<0.001	-0.007	0.001	-0.006	0.003	-0.007	0.001
mean BP, mmHg	-0.002(-0.005, 0.000)	0.072	-0.008(-0.012, -0.005)	<0.001	-0.008	<0.001	-0.006	0.001	-0.006	<0.001
Pulse pressure, mmHg	-0.001(-0.004, 0.001)	0.24	-0.005(-0.008, -0.002)	0.001	-0.004	0.037	-0.004	0.042	-0.004	0.030
Ea, mmHg/ml	-0.089(-0.136, -0.041)	<0.001	-0.12(-0.168, -0.072)	<0.001	-0.040	0.21	-0.034	0.27	-0.033	0.28
SVRI, dyne・m^2^/s・cm^⁻5^	-0.006(-0.009, -0.006)	<0.001	-0.010(-0.014, -0.006)	<0.001	-0.004	0.053	-0.004	0.098	-0.004	0.077
TAC, ml/mmHg	0.19(0.081, 0.290)	0.001	0.34(0.171, 0.512)	<0.001	0.17	0.059	0.15	0.082	0.15	0.086
LVMI, g/ m^2^	-0.002(-0.004, -0.001)	0.002	-0.004(-0.006, -0.002)	<0.001	-0.002	0.14	-0.001	0.40	-0.002	0.18
Ln baPWV, cm/sec⁑	-0.56(-1.172, 0.053)	0.072	-0.39(-1.158, 0.375)	0.29	-0.054	0.90	0.096	0.82	0.026	0.95
Ln E/e’(average), cm/sec										
systolic BP, mmHg	0.002(0.004, 0.943)	<0.001	0.005(0.003, 0.008)	<0.001	0.003	0.037	0.003	0.048	0.005	<0.001
diastolic BP, mmHg	0.00(-0.003, 0.003)	0.82	0.004(0.008, 0.860)	<0.001	0.004	0.093	0.004	0.13	0.004	0.086
mean BP, mmHg	0.001(-0.002, 0.004)	0.47	0.007(0.003, 0.011)	0.001	0.006	0.003	0.005	0.032	0.005	0.024
Pulse pressure, mmHg	0.004(0.001, 0.007)	0.007	0.007(0.003, 0.011)	<0.001	0.003	0.13	0.003	0.14	0.003	0.13
Ea, mmHg/ml	0.006(-0.048, 0.061)	0.82	0.058(-0.003, 0.120)	0.063	0.052	0.17	0.047	0.21	0.047	0.21
SVRI, dyne・m^2^/s・cm^⁻5^	0.002(-0.001, 0.004)	0.20	0.005(0.011, 0.766)	<0.001	0.003	0.34	0.002	0.47	0.002	0.41
TAC, ml/mmHg	-0.072(-0.192, 0.048)	0.24	-0.20(-0.412, 0.017)	0.071	-0.13	0.24	-0.067	0.27	0.11	0.30
LVMI, g/ m^2^	0.003(0.002, 0.005)	<0.001	0.003(0.001, 0.006)	0.009	0.003	0.98	0.00	0.86	0.00	0.93
Ln baPWV, cm/sec⁑	0.59(0.016, 1.163)	0.044	0.94(0.259, 1.618)	0.010	0.38	0.29	0.48	0.20	0.36	0.35
Ln LAVI max, ml/ m^2^										
systolic BP, mmHg	0.004(0.001, 0.006)	0.002	0.005(0.002, 0.007)	0.001	0.001	0.87	0.001	0.42	0.004	<0.001
diastolic BP, mmHg	0.00(-0.003, 0.003)	0.92	0.001(-0.004, 0.005)	0.79	0.001	0.57	0.001	0.65	0.002	0.49
mean BP, mmHg	0.002(-0.001, 0.005)	0.18	0.003(-0.001, 0.007)	0.088	0.004	0.072	0.002	0.50	0.002	0.43
Pulse pressure, mmHg	0.007(0.004, 0.010)	<0.001	0.009(0.005, 0.012)	<0.001	0.002	0.32	0.002	0.34	0.002	0.37
Ea, mmHg/ml	-0.063(-0.121, -0.004)	0.036	-0.067(-0.127, -0.007)	0.028	0.008	0.84	0.004	0.92	-0.001	0.99
SVRI, dyne・m^2^/s・cm^⁻5^	0.001(-0.002, 0.005)	0.53	-0.002(-0.008, 0.003)	0.38	-0.001	0.61	-0.002	0.48	-0.002	0.44
TAC, ml/mmHg	0.006(-0.125, 0.138)	0.92	0.044(-0.167, 0.256)	0.68	0.016	0.89	0.025	0.83	0.042	0.71
LVMI, g/ m^2^	0.007(0.005, 0.009)	<0.001	0.009(0.007, 0.011)	<0.001	0.002	0.19	0.002	0.20	0.002	0.19
Ln baPWV, cm/sec⁑	0.023(-0.601, 0.646)	0.94	0.38(-0.254, 1.005)	0.22	0.18	0.64	0.35	0.36	0.19	0.61
LAEF, %										
systolic BP, mmHg	0,.029(-0.059, 0.117)	0.52	-0.16(-0.249, -0.062)	0.001	0.001	0.001	-0.21	0.001	-0.18	0.001
diastolic BP, mmHg	-0.021(-0.152, 0.109)	0.75	-0.10(-0.250, 0.046)	0.18	-0.16	0.092	-0.17	0.082	-0.16	0.093
mean BP, mmHg	0.009(-0.110, 0.128)	0.89	-0.14(-0.280, 0.000)	0.050	-0.20	0.018	-0.22	0.013	-0.22	0.016
Pulse pressure, mmHg	0.077(-0.042, 0.197)	0.20	-0.23(-0.362, -0.104)	<0.001	-0.31	0.001	-0.31	0.001	-0.32	<0.001
Ea, mmHg/ml	-1.21(-3.541, 1.124)	0.31	-2.15(-4.267, -0.030)	0.047	0.89	0.56	-0.97	0.52	0.82	0.59
SVRI, dyne・m^2^/s・cm^⁻5^	-0.21(-0.346, -0.069)	0.004	-0.15(-0.336, 0.038)	0.12	-0.042	0.70	-0.058	0.59	-0.039	0.72
TAC, ml/mmHg	0.13(-5.028, 5.281)	0.96	4.56(-2.899, 12.01)	0.23	3.27	0.45	3.19	0.46	2.96	0.50
LVMI, g m^2^	-0.072(-0.141, -0.003)	0.040	-0.11(-0.202, -0.021)	0.016	-0.048	0.40	-0.53	0.36	-0.053	0.36
Ln baPWV, cm/sec⁑	6.73(-23.06, 36.51)	0.65	-9.40(-44.77, 25.96)	0.58	-7.63	0.71	-17.8	0.38	-10.20	0.62
LVEF, %										
systolic BP, mmHg	-0.035(-0.076, 0.006)	0.091	-0.005(-0.046, 0.036)	0.81	0.048	0.093	0.048	0.095	0.014	0.11
diastolic BP, mmHg	-0.060(-0.121, 0.000)	0.051	-0.014(-0.078, 0.049)	0.66	0.076	0.078	0.076	0.079	0.075	0.080
mean BP, mmHg	-0.059(-0.114, -0.004)	0.035	-0.020(-0.080, 0.040)	0.51	-0.004	0.026	0.066	0.10	0.068	0.085
Pulse pressure, mmHg	-0.017(-0.072, 0.039)	0.56	0.004(-0.053, 0.062)	0.88	-0.005	0.69	0.031	0.44	0.042	0.31
Ea, mmHg/ml	-1.70(-2.768, -0.629)	0.002	-0.61(-1.519, 0.302)	0.19	1.53	0.024	1.54	0.024	1.65	0.014
SVRI, dyne・m^2^/s・cm^⁻5^	-0.11(-0.172, -0.043)	0.001	-0.070(-0.150, 0.009)	0.084	0.086	0.074	0.088	0.071	0.097	0.045
TAC, ml/mmHg	2.94(0.568, 5.319)	0.015	2.34(-0.827, 5.514)	0.15	-1.71	0.38	-1.66	0.39	-2.96	0.28
LVMI, g/ m^2^	-0.042(-0.074, -0.010)	0.010	-0.019(-0.058, 0.020)	0.33	0.018	0.48	0.016	0.53	0.023	0.37
Ln baPWV, cm/sec⁑	2.39(-11.80, 16.58)	0.73	-2.28(-12.79, 8.229)	0.65	-6.53	0.43	-8.31	0.33	-6.38	0.46
s’(average), cm/sec										
systolic BP, mmHg	-0.007(-0.020, 0.005)	0.24	-0.017(-0.029, -0.005)	0.006	-0.009	0.30	-0.009	0.30	-0.016	0.033
diastolic BP, mmHg	0.001(-0.017, 0.020)	0.88	-0.017(-0.035, 0.002)	0.073	-0.017	0.19	-0.017	0.20	-0.017	0.19
mean BP, mmHg	-0.006(-0.023, 0.011)	0.50	-0.023(-0.040, -0.005)	0.011	-0.021	0.059	-0.016	0.20	-0.015	0.20
Pulse pressure, mmHg	-0.014(-0.031, 0.002)	0.094	-0.019(-0.035, -0.002)	0.029	-0.005	0.69	-0.005	0.70	-0.005	0.66
Ea, mmHg/ml	-0.25(-0.578, 0.080)	0.14	-0.21(-0.475, 0.061)	0.13	0.11	0.61	0.11	0.59	0.11	0.60
SVRI, dyne・m^2^/s・cm^⁻5^	-0.041(-0.060, -0.022)	<0.001	-0.033(-0.056, -0.010)	0.005	0.010	0.46	0.011	0.46	0.010	0.51
TAC, ml/mmHg	0.67(-0.057, 1.391)	0.071	0.77(-0.167, 1.697)	0.11	-0.067	0.91	-0.073	0.90	-0.076	0.90
LVMI, g/ m^2^	-0.013(-0.022, -0.003)	0.011	-0.014(-0.026, -0.003)	0.013	-0.004	0.63	-0.004	0.61	-0.004	0.64
Ln baPWV, cm/sec⁑	-0.958(-4.543, 2.628)	0.59	-1.527(-4.452, 1.399)	0.28	0.74	0.72	-0.78	0.72	-0.69	0.75

Above multivariable models were adjusted for height, weight, DM, IHD, smoking, Hb and Cr.

e’, end diastolic velocity of the mitral annulus; LAVI max, maximum left atrial volume index; LAEF, left atrial emptying fraction; LVEF, left ventricular ejection fraction; s’, systolic mitral annular velocity; BP, blood pressure; Ea, arterial elastance; SVRI, systemic vascular resistance index; TAC, total arterial compliance; LVMI, left ventricular mass index; Ln baPWV, log-transformed brachial ankle pulse wave velocity.

⁑In whole population, baPWV was measured in 69 cases (44 in men, in 25 women). In age matched population, 49 cases (25 in men, 24 in women)

Among the subgroup of patients with baPWV (n = 69, male; 44, female; 25), we observed similar associations: significant relationship between baPWV and e’ in women but not in men. However, the interaction was not statistically significant (p = 0.19), presumably due to less statistical power. In addition, the strongest determinants of the aortic size were BSA for the aortic length and age for the aortic volume (Table 6).

**Table 6:** The association with aorta size (Aortic length and volume).

	Aortic Length(R^2^ = 0.50)		Aortic Volume(R^2^ = 0.44)	
Age, years	0.21(0.137, 0.280)	<0.001	2.49(1.524, 3.460)	<0.001
Body surface area, m^2^	12.0(7.892, 16.07)	<0.001	146.3(204.7, 204.7)	<0.001
Ln E/e’(average)	-3.24(-5.888, -0.588)	0.017	-	-
Diabetes	-	-	-21.7(-43.05, -0.445)	0.045

Above multivariable regression analysis were adjusted for age, body size(height, weight, BSA and BMI), coronary risk factor(HT,DM,DL,IHD), afterload parameters(systolic BP, diastolic BP, mean BP, Pulse Pressure, Ea, SVRI, TAC, LVMI and baPWV) and parameters of cardiac function(Ln e’, Ln E/e’, Ln LAVI, LAEF, LVEF and s’).

## Discussion

This is the first study which assessed whether the aortic size alters the magnitude of sex difference between LV diastolic function and afterload. Through a comprehensive hemodynamic evaluation of elderly patients with preserved EF, we found 1) that only weak associations were found between BSA and the aortic sizes (length and volume) measured with CT; 2) that we confirmed statistically significant sex differences in the relationships between LV diastolic function and afterload in our population; 3) that adjusting for the aortic sizes had minimal effects on the sex dimorphism; 4) that sex differences were independent from the aortic sizes, more accurate anatomical parameters of the vascular sizes; and 5) that among the afterload parameters, aggressive BP lowering would be more beneficial in women than in men because sex difference was more prominent between diastolic function and BPs.

### Sex differences between LV diastolic function and afterload

Although male sex is a risk factor for many cardiovascular diseases, women outnumber men with HFPEF by a 2:1 ratio.[23, 24] Previous studies have shown sex differences between LV diastolic function and afterload in smaller populations with different perspectives.[8, 19] Shim et al. reported a sex disparity in the relationship between PP amplification and e′ or E/e′ among 158 age-matched subjects (79 men and 79 women).[8] Coutinho et al. demonstrated a sex difference in the association between the E/A ratio and afterload (i.e., TAC or aortic impedance) among 461 non-heart failure participants.[19] Our study corroborated these findings in a larger sample of non-heart failure population with preserved EF in order to avoid the contaminating effects from reduced EF, where women had slower LV relaxation (e′), higher filling pressure (E/e′), and lower LA function (LAEF). This was supported by higher serum BNP levels in women than in men. Intriguingly, there were discrepancies among these diastolic functional parameters between the sexes. Statistically significant interactions by sex were detected in functions but not in LA size. This could be due to our cross-sectional study design, because LA volume reflects chronic LA pressure overload and would be less sensitive to instantaneous changes in hemodynamics.[25]

In this study of elderly people with preserved EF, although BPs were similar between the sexes, women had greater afterload (Ea and TAC) as well as more deteriorated diastolic function. This finding supports a previous report that advanced age and female sex were associated with increases in vascular and ventricular stiffness, even in the absence of cardiovascular diseases.[26] Furthermore, we focused on elderly people because aging is an independent risk factor for both types of HF[27]. However, it is noted that a southwestern European community study reported that the prevalence of HFPEF increased more rapidly with age than did the prevalence of HFREF.[6]

Next, there were two intriguing findings on LVEF in this study. A greater LVEF was observed in women, consistent with a report from the Dallas Heart Study,[28] which also suggested different normal ranges for LVEF. The other finding was that associations between LVEF and afterload were only significant among men. In other words, there were no relationships between LVEF and afterload markers in women, suggesting that LVEF may not be a sensitive parameter of afterload burden in women. Collectively, a greater impairment in diastolic function among elderly women with increased afterload may possibly contribute to greater susceptibility to HFPEF, though further study is warranted.

This was the first study to focus on the aortic length and volume as measured by CT in order to determine whether there was significant effect modification by sex on the effects of afterload on LV diastolic function even after adjusting for the disparity in body size by sex. Previous studies have tested independent associations after adjusting for body surface area (BSA), based on the assumption that BSA is a reasonable surrogate for aortic length[7, 8] although little is known about the strength of the association between BSA and aortic volume/length. Furthermore, BSA is only one of the factors affecting the aortic size.[29, 30] Indeed, BSA and aorta size had only weak association in both men and women in this study. Thus, we thought it is more reasonable adjusting for the aorta sizes considering the mechanism underlying the arterial wave reflections and function of buffering pulsatile pressure from the heart. [31] Because sex difference between LV diastolic function and afterload remained significant after adjusting for aorta size, the differences in the conduit size did not fully account for the sex difference in the relationship between afterload and LV relaxation.

### Study limitations

Some limitations should be noted for this study. Firstly, this was a retrospective, cross-sectional study performed in a single tertiary institution and in a population with a single ethnicity. It therefore may have inherent flaws related to selection bias, spurious observations, and unmeasured covariates. Due to the retrospective nature of the data collection, several pieces of information were not available, including history of physical exercise and precise reason/s for BNP measurements. Thus, further prospective, multicenter and multi-ethnicity studies are warranted. Secondly, we calculated hemodynamic parameters with BP measured at a peripheral artery, where these parameters might be different from true central hemodynamics.[32, 33] The lack of a central hemodynamic data might make these results linked to a high variability. However, sex differences were the most frequently found in BPs. Thirdly, we used the aortic sizes as vascular surrogate of the body size related reflected wave, but we did not measure reflected wave itself. Therefore, we couldn’t elucidate directly that gender difference was associated with reflected wave. However, we observed similar association between e’ and baPWV, where only women had a significant association but this was not seen in men although the interaction was not significant due to smaller sample size (n = 69) and less statistical power. Also, because we excluded patients with atrial fibrillation, our results cannot be readily applicable to those patients. In addition, although our patients’ body sizes were normal for Asians, our population had a smaller overall body size (mean body mass index, 22.4± 3.8kg/m^2^) than that in Western populations.[19, 34] Nevertheless, our findings on the relationship between afterload and diastolic function were generally similar to those reported in past studies using patients with larger body sizes.[19, 34] Furthermore, the population of this study were not normal but aged and diseased samples, who had clinical indications for taking CT and echocardiography. They might not reflect the normal physiology; however, this means they were at Stage A or B HF patients, which would be a reasonable target to be investigated because of the risk of subsequent obvert HF. Finally, half of the CT images were performed without contrast, which may limit the accuracy of the aortic volume assessment, but the effect should be minimal on the aortic length, where most of the findings were consistent between the volume and length. Despite these limitations, this is the first report to show significant sex differences between diastolic function and afterload before and after adjusting for the aortic sizes.

## Conclusions

Significant sex differences in the relationships between LV diastolic functions and afterload were confirmed in elderly patients with preserved ejection fraction. Women had worse LV relaxation compared to those in men with the same degree of afterload, before and after adjusting for the aortic sizes.

## Abbreviations

baPWV: brachial-ankle pulse wave velocity
BNP: brain natriuretic peptide
BSA: body surface area
CT: computed tomography
DBP: diastolic blood pressure
Ea: arterial elastance
HF: heart failure
HFPEF: heart failure with preserved ejection fraction
HFREF: heart failure with reduced ejection fraction
LAEF: left atrial emptying fraction
LAVI: left atrial volume index
LV: left ventricular
LVEF: left ventricular ejection fraction
PP: pulse pressure
BP: systolic blood pressure
SV: systolic volume
SVRI: systemic vascular resistance index
TAC: total arterial compliance
